# The Medical Profession and the Uncertified Midwife

**Published:** 1917-06-02

**Authors:** 


### THE MEDICAL PROFESSION AND THE UNCERTIFIED MIDWIFE.

During the recent session of the General Medical Council cases have been considered in which the Central Midwives Board has complained of the conduct of individual medical men who have, as it is alleged, "covered" the practice of unregistered midwives. The General Medical Council, in some at least of these cases, has adjudged the medical men thus summoned to be guilty of "infamous conduct in a professional respect," has severely admonished the offenders, and has punished them by requiring them to attend again in six months' time with testimonials as to their behaviour in the meanwhile.

With the "covering" of unqualified and unregistered practice of any kind, as with all sorts of fraud and quackery, The Hospital has not and never has had any sympathy; and those who act so unethically and so contrary to the best interests of the public health deserve all the pains and penalties (usually quite inadequate) which the law allows to be inflicted upon them. We will grant for the sake of our present argument the supposition, since it is broad principles alone and not any individual case to which we would draw attention, that the medical men recently haled before the General Medical Council at the instance of the Central Midwives Board were, in fact, conniving at or actively assisting the practice of unregistered midwives, and were guilty by intention of the offence of "covering." That does not by any means exhaust the situation, however. Medical men in various parts of the country are complaining that some County Medical Officers of Health (the local authorities under the Midwives Act) have written what are in effect threatening letters. These letters have in certain instances begun by drawing the attention of the practitioner to the fact that he has attended in an emergency a case of labour until then conducted by an unregistered woman, and have continued with the explicit threat that any similar occurrence in future will be made the subject of a complaint to the General Medical Council. It is further believed that it is the Central Midwives Board which has forced the County Medical Officers of Health into what is to the latter, doubtless, a distasteful business.

If these threats and warnings had been addressed solely to practitioners of doubtful integrity or of blemished reputation, men suspected on reasonable grounds of deliberately "covering" for gain unregistered midwives, there would be little to say about the matter. But they have, unless our information is faulty, been addressed to several men of the highest reputation, whose abhorrence of "covering" is just as genuine as that of the General Medical Council itself. And the effect of obedience to these behests would simply be that medical men would be estopped from giving their skill to difficult cases of labour whenever unregistered midwives had charge of the cases; and that the lives of a certain number of mothers and children would be sacrificed as a direct consequence. Public opinion —and professional opinion too—would rightly make things warm for a doctor whose refusal to attend an obstetric emergency on the ground that the midwife already in attendance was unregistered resulted in the death of mother or child; it is not difficult to imagine what a coroner's jury would say to such a plea. Deliberate "covering" should be stopped; but this is not "covering" in the true sense of that word, and if the Central Midwives Board or the General Medical Council should so interpret it, those bodies will soon find themselves badly out of touch with popular opinion. In many hamlets and villages there is no such thing as a registered midwife available; and if medical men are not to be allowed, under pain of disregistration, to go to the help of parturient women in such places unless they have been duly engaged beforehand, a very real grievance and hardship will undoubtedly be created.

